# Integrating transcriptome-wide association study and mRNA expression profile identified candidate genes related to hand osteoarthritis

**DOI:** 10.1186/s13075-021-02458-2

**Published:** 2021-03-10

**Authors:** Jiawen Xu, Yi Zeng, Haibo Si, Yuan Liu, Mingyang Li, Junfeng Zeng, Bin Shen

**Affiliations:** Department of Orthopaedic Surgery, West China Hospital, West China Medical School, Sichuan University, 37# Guoxue Road, Chengdu, 610041 Sichuan Province People’s Republic of China

**Keywords:** Hand osteoarthritis, Transcriptome-wide association study (TWAS), mRNA expression profile, Gene ontology (GO), Pathway enrichment analysis

## Abstract

**Background:**

Osteoarthritis (OA) is a common skeletal system disease that has been partially attributed to genetic factors. The hand is frequently affected, which seriously affects the patient’s quality of life. However, the pathogenetic mechanism of hand osteoarthritis (hand OA) is still elusive.

**Methods:**

A genome-wide association study (GWAS) summary of hand OA was obtained from the UK Biobank dataset, which contains data from a total of 452,264 White British individuals, including 37,782 OA patients. The transcriptome-wide association study (TWAS) of hand OA was performed using FUnctional Summary-based ImputatiON (FUSION) with the skeletal muscle and blood as gene expression references. The significant genes identified by TWAS were further subjected to gene set enrichment analysis (GSEA) with the Database for Annotation, Visualization and Integrated Discovery (DAVID) tool. Furthermore, we compared the genes and gene sets identified by our TWAS with that of a knee OA mRNA expression profile to detect the genes and gene sets shared by TWAS and mRNA expression profiles in OA. The mRNA expression profiles of 18 normal knee cartilages and 20 OA knee cartilages were acquired from the Gene Expression Omnibus database (accession number: GSE114007).

**Results:**

TWAS identified 177 genes with *P* < 0.05 for the skeletal muscle, including ANKRD44 (*P* = 0.0001), RIC3 (*P* = 0.0003), and AC005154.6 (*P* = 0.0004). TWAS identified 423 genes with *P* < 0.05 for the blood, including CRIM1 (*P* = 0.0002), ZNF880 (*P* = 0.0002), and NCKIPSD (*P* = 0.0003). After comparing the results of the TWAS to those of the mRNA expression profiling of OA, we identified 5 common genes, including DHRS3 (log2fold = − 1.85, *P* = 3.31 × 10^− 9^) and SKP2 (log2fold = 1.36, *P* = 1.62 × 10^− 8^). GSEA of TWAS identified 51 gene ontology (GO) terms for hand OA, for example, protein binding (*P* = 0.0003) and cytosol (*P* = 0.0020). We also detected 6 common GO terms shared by TWAS and mRNA expression profiling, including protein binding (*P*_TWAS_ = 2.54 × 10^− 4^, *P*_mRNA_ = 3.42 × 10^− 8^), extracellular exosome (*P*_TWAS_ = 0.02, *P*_mRNA_ = 1.18 × 10^− 4^), and cytoplasm (*P*_TWAS_ = 0.0183, *P*_mRNA_ = 0.0048).

**Conclusion:**

In this study, we identified 5 candidate genes and 6 GO terms related to hand OA, which may help to uncover the pathogenesis of hand OA. It should be noted that the possible difference in the gene expression profiles between hand OA and knee OA may affect our study results, which should be interpreted with caution.

**Supplementary Information:**

The online version contains supplementary material available at 10.1186/s13075-021-02458-2.

## Introduction

Osteoarthritis (OA) is a kind of musculoskeletal system disease that occurs in many elderly individuals. The hand is one of the locations where OA occurs most often, especially in women. The main symptoms are joint stiffness, pain, and even swelling. OA affects up to 10% of men and 13% of women worldwide [1]. Among women aged 60 to 70 in the USA, the proportion of women suffering from hand OA is as high as 75% [2]. The hand flexion and extension activity of hand OA patients is seriously affected, and hand function and quality of life are reduced as a result. With the aging of society, an increasing number of hand OA patients may be seen in the future.

With the development of bioinformatics, an increasing number of studies have focused on the genetic underpinnings of OA. At the genetic level, there seem to be several mechanisms that may differ between hand OA and knee or hip OA [3]. Through genome-wide association studies (GWASs), researchers have found several loci significantly associated with OA, such as chr12 near the matrix Gla protein (MGP) gene and chr12 near the CCDC91 gene [4]. Several significant functional polymorphisms were also found between hand OA patients and normal subjects, such as ASPN D15 and CILP rs2073711 TT [5]. Unfortunately, the genetic mechanism underlying hand OA remains unclear.

GWASs have been successfully applied for gene mapping of complex human diseases and traits. However, this approach is limited in evaluating disease risk, because most GWAS-identified SNPs are located in non-coding regions of the genome. Variant SNPs may play a role in gene expression levels [6]. Expression quantitative trait loci (eQTL) analysis aims to identify genes related to variation in gene expression [7]. Therefore, integrating GWAS and eQTL analysis helps researchers to identify genes with causal associations to disease more effectively. The unique advantages of transcriptome-wide association studies (TWASs) have gradually been highlighted in recent years. TWAS combines the precomputed gene expression weights with summary statistical data from GWAS to recognize novel causal genes of elusive diseases [8]. In recent years, TWASs have been utilized by an increasing number of researchers to identify genetic loci associated with the disease. For example, a TWAS of approximately 3000 subjects found 69 novel genes significantly related to BMI, lipid profile, and height that have a bearing on obesity [9]. A TWAS investigating chronic low-grade inflammation recognized 448 genes related to inflammatory biologic age [10].

**Fig. 1:**
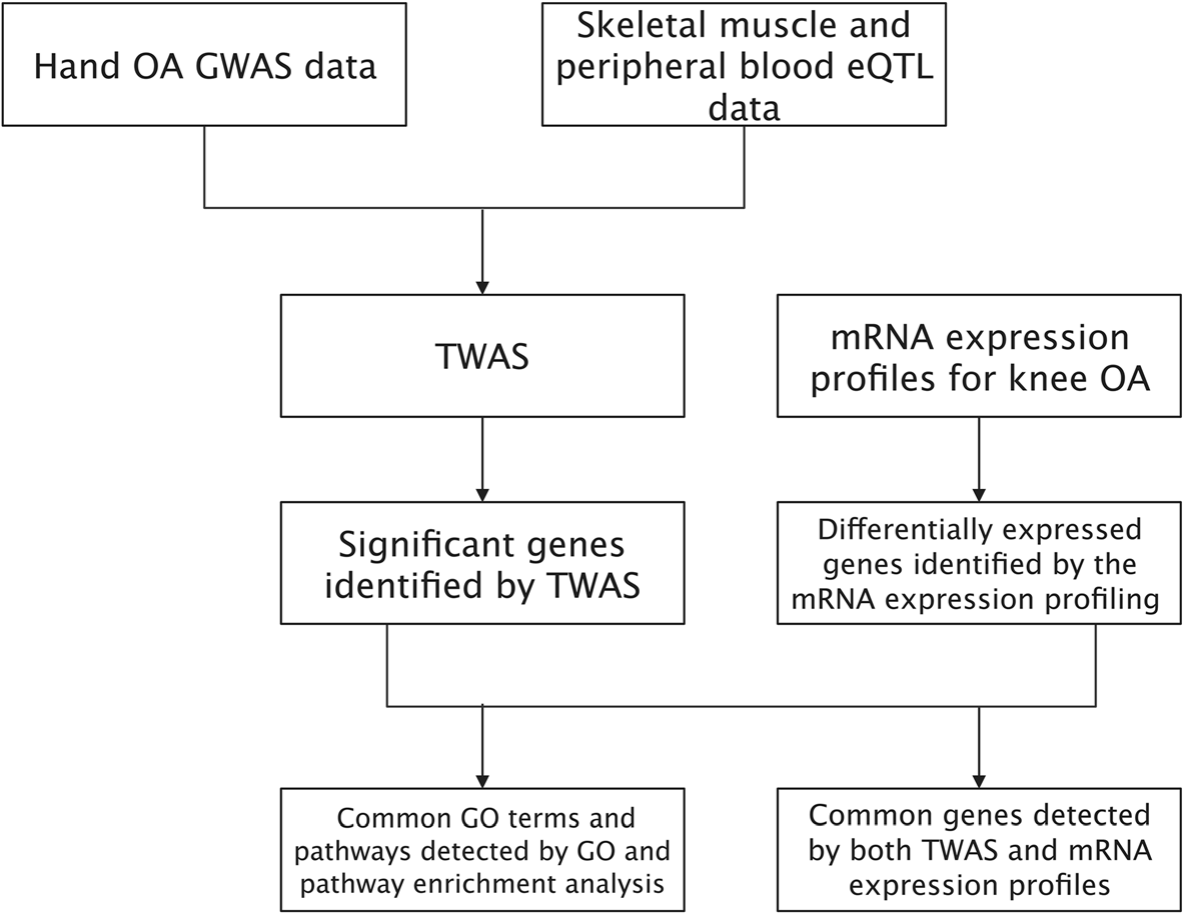
Detailed flowchart of our study design. GWAS genome-wide association study, eQTL expression quantitative trait locus, TWAS transcriptome-wide association study, GO gene ontology

In this study, we used a large GWAS dataset for hand OA in conjunction with cartilage mRNA expression profiling. A TWAS was performed first to find genetic loci that may be associated with hand OA. Then, gene ontology (GO) and pathway enrichment analysis were carried out for the notable genes screened by TWAS. To identify common genes and biological pathways, the significant genes identified by the TWAS were compared with the OA profile analysis results, as well as GO and pathway analyses (Fig. 1). It should be noted that the difference in the gene expression profiles between hand OA and knee OA may affect our study results. More biological studies should be conducted to confirm our study results.

## Material and method

### GWAS summary datasets for hand OA

The GWAS summary data for hand OA was derived from the UK Biobank samples (UK Biobank fields: 20002) [11]. Briefly, the UK Biobank cohort contains 452,264 White British individuals, including 37,782 osteoarthritis patients [11]. Information about various phenotypes has been collected from every participant, and blood samples were taken when the subjects visited a UK Biobank assessment center. DNA extraction and genotyping were performed in the Affymetrix Research Services Laboratory. There were 62,394 genotyped variants that passed quality control through the Applied Biosystems UK Biobank Axiom Array. This dataset contains 9,113,133 imputed variants after filtering. The IMPUTE4 program was used to perform the imputation (http://jmarchini.org/software/). Detailed information on the subjects, genotyping, imputation, and quality control can be found in the published study [11].

### Gene expression profiles of cartilage

The mRNA expression profiles of knee OA were acquired from the Gene Expression Omnibus database (accession number: GSE114007). Briefly, the normal human knee cartilage tissues were collected from 5 females and 13 males (age 18–61, mean 38), who had never suffered from joint disease or trauma and processed within 24–48 h postmortem. The knee cartilage specimens affected by OA were gathered from 12 females and 8 males undergoing knee replacement surgery (age 52–82, mean 66). Body mass indices between the normal controls (BMI = 32.4 ± 8.0) and OA patients (BMI = 30.7 ± 8.1) were not significantly (*p* = 0.506) different [12]. The original image data were transformed into sequence data by the Illumina Genome Analyzer Pipeline Software (Casava v1.8.2). Genes designated as expressed in the study were those whose log counts per million (log2CPM) were greater than 3.0 in one or more samples. Limma-voom was carried out for differential expression analysis. Genes were designated as significantly differentially expressed (DE) when the following two conditions were met: adjusted *P* value of < 0.05 by the moderated *t* statistic and |log2FC| > 1 [12].

### TWAS of hand OA

The TWAS of hand OA was carried out using the FUnctional Summary-based ImputatiON (FUSION) approach (http://gusevlab.org/projects/fusion/) [9]. Given that the skeletal muscle and peripheral blood were used in previous biological studies of OA [13, 14], the FUSION precomputed functional weights of gene expression of the skeletal muscle and peripheral blood were used in our TWAS of hand OA. In brief, we took advantage of the prediction models implemented in FUSION to calculate the gene expression. Then, the calculated tissue-related expression weights were integrated with summary-level GWAS results to impute the association statistics between gene expression and the target disease. In this study, the gene expression weight panels for the peripheral blood and skeletal muscle were downloaded from the FUSION website (http://gusevlab.org/projects/fusion/).

### GO and pathway enrichment analysis

The candidate genes identified by TWAS were further analyzed by the Database for Annotation, Visualization and Integrated Discovery (DAVID, https://david.ncifcrf.gov/) tool for GO and pathway enrichment analysis. Similarly, DAVID was also applied to the differentially expressed genes identified by the mRNA expression profiling of OA cartilage. To find the common GO and pathways detected both by TWAS and mRNA expression profiles, we carefully compared the DAVID analysis results of TWAS and cartilage mRNA expression profiles.

## Results

### TWAS results of hand OA

The TWAS identified 177 genes with a *P* value < 0.05 for the skeletal muscle, including ANKRD44 (*P* = 0.0001), RIC3 (*P* = 0.0003), and AC005154.6 (*P* = 0.0004). TWAS identified 423 genes with a *P* value < 0.05 for the blood, including CRIM1 (*P* = 0.0002), ZNF880 (*P* = 0.0002), and NCKIPSD (*P* = 0.0003) (Supplementary Table S1). The top 10 most significant genes identified by TWAS are shown in Table 1.

**Table 1:** Top 10 genes selected by TWAS analysis

Tissue	Gene name	Chromosome	TWAS.Z	TWAS.P
Muscle skeleton	ANKRD44	2	3.8381	0.0001
	RIC3	11	3.6172	0.0003
	AC005154.6	7	3.5112	0.0004
	PARD3	10	3.4069	0.0007
	GGCT	7	3.3363	0.0008
Peripheral blood	CRIM1	2	−3.6781	0.0002
	ZNF880	19	3.6694	0.0002
	NCKIPSD	3	−3.629	0.0003
	CCR3	3	−3.5323	0.0004
	R3HCC1	8	3.5037	0.0005

Note: The GWAS summary data for hand OA was derived from the UK Biobank (UK Biobank fields: 20002), which contains 452,264 White British individuals, including 37,782 osteoarthritis patients. The TWAS. P and TWAS. Z values were calculated by the FUSION approach (http://gusevlab.org/projects/fusion/)

*TWAS* Transcriptome-Wide Association Study, *GWAS* Genome-Wide Association Study, *TWAS* P TWAS *P* value, *TWAS* Z TWAS Z score

### GO and pathway analysis results of TWAS

DAVID analysis of TWAS identified 51 GO terms enriched in hand OA, such as protein binding (*P* value = 2.54 × 10^− 4^), cytosol (*P* value = 0.0012), and membrane (*P* value = 0.0014). We also detected 5 biological pathways for hand OA, including biosynthesis of unsaturated fatty acids (*P* value = 0.0034) and propanoate metabolism (*P* value = 0.0071) (Supplementary Table S2, S3).

### Integrative analysis of TWAS and mRNA expression profiling of OA

A comparison of the genes identified by TWAS with the differentially expressed genes detected by mRNA expression profile yielded 5 common genes: DHRS3 (log2fold = − 1.85, *P* value = 3.31 × 10^− 9^), SKP2 (log2fold = 1.36, *P* value = 1.62 × 10^− 8^), IRS2 (log2fold = − 2.00, *P* value = 2.11 × 10^− 9^), TOB1 (log2fold = − 2.67, *P* value = 8.42 × 10^− 9^), PPP1R15A (log2fold = − 2.13, *P* value = 2.83 × 10^− 8^) (Table 2).

**Table 2:** The candidate genes identified by both TWAS and mRNA expression profiles of osteoarthritis

Gene name	Chromosome	TWAS.Z	TWAS.P	Log2fold change
DHRS3	1	−2.699	0.0070	−1.8451
SKP2	5	1.9814	0.0475	1.3556
IRS2	13	−2.130	0.0331	2.0001
TOB1	17	1.9970	0.0458	−2.6653
PPP1R15A	19	2.2334	0.0255	−2.1270

Note: Using the GWAS summary data of hand osteoarthritis (OA), transcriptome-wide association study (TWAS) was conducted using the FUSION approach. The mRNA expression profiles of knee OA was acquired from the Gene Expression Omnibus database (accession number: GSE114007). The common candidate genes were finally detected by comparing the significant genes identified by TWAS with the differently expressed genes detected by the mRNA expression profiles

*TWAS* Transcriptome-Wide Association Study, *TWAS P* TWAS *P* value, *TWAS Z* TWAS *Z* score

A comparison of the GO enrichment analysis results of TWAS and mRNA expression profiles yielded 6 common GO terms: protein binding (*P*_TWAS_ = 2.54 × 10^− 4^, *P*_mRNA_ = 3.42 × 10^− 8^), extracellular exosome (*P*_TWAS_ = 0.0225, *P*_mRNA_ = 1.18 × 10^− 4^), cytoplasm (*P*_TWAS_ = 0.0183, *P*_mRNA_ = 0.0048), oxidoreductase (*P*_TWAS_ = 0.0312, *P*_mRNA_ = 0.0259), cellular response to mechanical stimulus (*P*_TWAS_ = 0.0324, *P*_mRNA_ = 0.0053), and oxidation-reduction process (*P*_TWAS_ = 0.0424, *P*_mRNA_ = 0.0294) (Table 3).

**Table 3:** GO terms identified by both TWAS and mRNA expression profiles of osteoarthritis

GO term	*P* value for TWAS	*P* value for mRNA expression profile
GO:0005515 ~ protein binding	0.0003	3.42E−08
GO:0005737 ~ cytoplasm	0.0183	0.0048
GO:0070062 ~ extracellular exosome	0.0225	0.0001
GO:0016491 ~ oxidoreductase activity	0.0312	0.0259
GO:0071260 ~ cellular response to mechanical stimulus	0.0323	0.0053
GO:0055114 ~ oxidation-reduction process	0.0424	0.0294

Note: the candidate genes identified by TWAS and mRNA expression profiles were further analyzed by the Database for Annotation, Visualization and Integrated Discovery tool (https://david.ncifcrf.gov/) for GO enrichment analysis, respectively. The common GO terms were finally detected by comparing the significant GO terms identified for TWAS the mRNA expression profiles

*GO* gene ontology, *mRNA* messenger RNA, *TWAS* Transcriptome-Wide Association Study

## Discussion

Our study aimed to detect candidate genes closely associated with hand osteoarthritis and to explain the relationships between these genes and the disease. We conducted a TWAS and functional gene set enrichment analysis and identified multiple genes and enriched GO terms and pathways for hand OA. Further integrative analysis of TWAS and mRNA expression profiling of OA identified 5 common genes and 6 common GO terms shared by TWAS and the mRNA expression profile of OA. Our study results provide novel clues for understanding the genetic mechanism of hand OA, focusing on the roles of abnormal gene transcription in the development of OA.

**Fig. 2:**
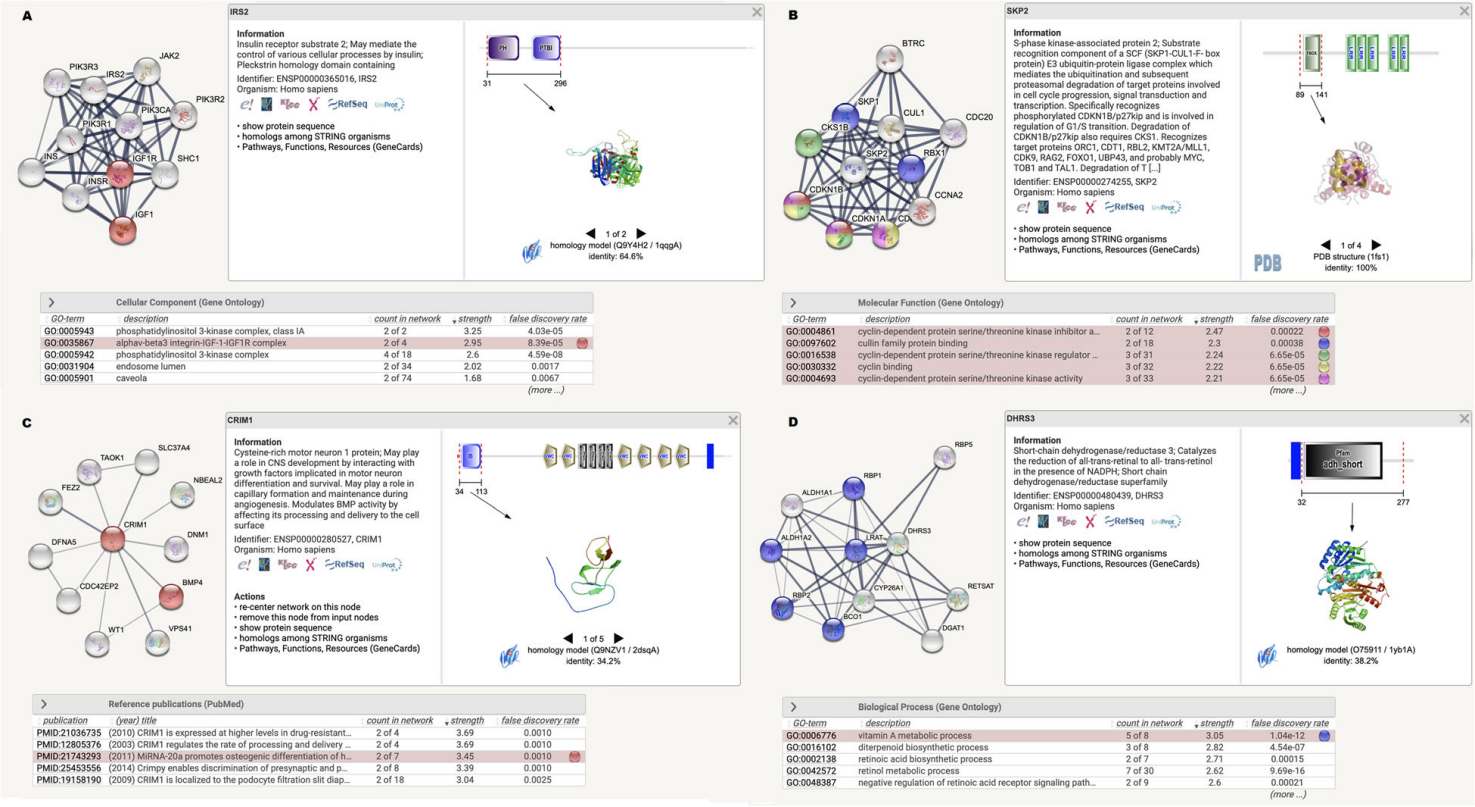
Image showing protein-association networks of identified genes (**a** IRS2, **b** SKP2, **c** CRIM1, **d** DHRS3) for osteoarthritis. The image was generated using the STRING online tool (https://www.string-db.org/). The enriched function which is associated with the mechanism of osteoarthritis has been selected during the analysis, and the corresponding proteins nodes in the network are automatically highlighted in color

TWAS identified several candidate genes for hand OA, such as IRS2, SKP2, CRIM1, and DHRS3 (Fig. 2). IRS2 (insulin receptor substrate 2) is a cytoplasmic adaptor molecule. It encodes some essential proteins related to insulin transduction and indeed plays a role in insulin resistance [15]. Insulin-like growth factor 1 (IGF1) has important effect on bone growth and cellular differentiation. IGF1 is highly expressed in the chondrocytes and can promote the proliferation of chondrocytes. A previous study proved that IRS2 could influenced chondrocyte differentiation by targeting RUNX1 to control signaling downstream of IGF1 [16].

SKP2 was also detected in both the TWAS and mRNA expression profiles of OA. SKP2 gene encodes S-phase kinase-associated protein 2 (Skp2), which acts as an essential regulator of cell cycle. Previous study indicated that Skp2 also played a direct role in cellular senescence [17]. The prevalence of OA is positively correlated with age. The relationship between cellular aging and OA has recently been well investigated [18–20]. In addition, Skp2 is an important molecule that mediates the ubiquitination and degradation of p27 protein. Skp2/p27 pathway is a critical mediator of ER stress-induced growth arrest. ER stress is implicated in the development of OA [21].

CRIM1 gene encodes human cysteine-rich motor neuron protein 1. Researchers have found that the extracellular domain of CRIM1 could bind to bone morphogenetic protein (BMP) [22]. It has been found that chondrocyte hypertrophy could be inhibited by depressing BMP signaling pathway [23]. In addition, previous studies suggested that BMP was involved in skeletal morphogenesis and chondrogenesis and acted as a potential chemo-protective mediators that regulated matrix metabolism [24].

Another identified candidate gene for OA is DHRS3. DHRS3 gene encodes dehydrogenase reductase 3 enzyme [6, 25]. This enzyme is thought to play a role in regulating all-trans-retinaldehyde in the human body [6]. DHRS3 is detect in the biology process of vitamin A that plays an important role in bone and cartilage metabolism, and its deficiency is implicated in the pathological process of OA [26]. In a study of rheumatoid arthritis (RA), DHRS3 was detected as one candidate genes for RA, as its expression was twice as high in the RA case group as in the control group [27]. A previous study also found that DHRS3 mediated IL-1β-administered chondrocyte proliferation, apoptosis and ECM degradation by positively regulating GREM1 expression via competitively targeting miR-183-5p [28].

GO enrichment analysis was conducted to explore the functions of significant genes and how they are distributed in hand OA. In our study, oxidoreductase activity was identified by both TWAS and mRNA expression profiling. Previous research has shown that oxidoreductase activity is negatively related to collagen synthesis in osteoblasts [29]. Studies have also indicated that osteoblast dysregulation is strongly associated with OA pathogenesis [30]. Combined with the results of our study, these findings suggest that oxidoreductase activity may play a role in the mechanism of OA.

The oxidation-reduction process has been proven to be closely related to human aging and OA occurs more frequently in the elderly [31]. However, the mechanism requires further research.

Biosynthesis of unsaturated fatty acids is one pathway identified as enriched in OA by TWAS. Its function in OA has been investigated in many studies. For example, oleic acid is an unsaturated fatty acid, and previous research has indicated that it could relieve OA symptoms by controlling inflammation [32]. Eicosapentaenoic acid and docosahexaenoic acid are also unsaturated fatty acids and were both identified as substances that could reduce inflammation [33].

TWAS also detected the E2F1 destruction pathway as associated with hand OA. E2F1 is a transcription factor that has a critical function in controlling cell proliferation [34]. Previous research on osteoarthritis shows that E2F1 could boost osteoclastogenesis and induce inflammation [35]. The E2F1 destruction pathway may be a powerful therapeutic prospect for OA.

In this study, we compared the results of TWAS and mRNA expression profiling. Hand OA TWAS can detect genes with significant disease association at the DNA level, and mRNA expression profiling could provide information to explain the regulatory mechanisms of OA pathogenesis at the expression level. The combination of TWAS and mRNA expression profiling contributes to the more accurate identification of candidate genes.

Some limitations of this study should be noted. First, the GWAS summary data came from the UK Biobank cohort, in which almost all participants were of European ancestry. Therefore, it should be cautious to apply our study results to other populations. Second, to validate the TWAS results, we compared the significant genes identified by TWAS of hand OA with the differently expressed genes detected by the mRNA expression profiles of knee OA. The potential difference in the gene expression profiles between hand OA and knee OA may affect our study results, which should be interpreted with caution. Further biological studies should be conducted to confirm our findings.

## Conclusion

In summary, we integrated the GWAS datasets of hand OA from the UK Biobank and precomputed gene expression weights of the peripheral blood and skeletal muscle to complete the TWAS. Then, we selected genes that were significantly differentially expressed in OA cases. We also compared the results of GO and pathway enrichment analyses at the gene and transcription levels. Five common genes and 6 GO terms were selected. We hope that our results will further the study of the mechanism of hand OA at the genetic and molecular levels.

## Supplementary Information

**Additional file 1: Supplementary Table1**. TWAS results of hand osteoarthritis.

**Additional file 2: Supplementary Table2**. GO enrichment analysis results of candidate genes detected by TWAS of hand osteoarthritis.

**Additional file 3: Supplementary Table3.** Pathway enrichment analysis results of candidate genes identified by the TWAS of hand osteoarthritis.

## Data Availability

The datasets analyzed during the current study are available from the Gene Expression Omnibus database (https://www.ncbi.nlm.nih.gov/gds) accession number: GSE114007; the UK biobank (http://geneatlas.roslin.ed.ac.uk/) fields: 20002.

## Abbreviations

DAVID: Database for Annotation, Visualization and Integrated Discovery
DE: Different expression
eQTL: Expression quantitative trait loci
FUSION: Functional Summary-Based Imputation
GSEA: Gene set enrichment analysis
GO: Gene ontology
GWAS: Genome-wide association study
OA: Osteoarthritis
TWAS: Transcriptome-wide association study
