# Supplementary material for: Integrating transcriptome-wide association study and mRNA expression profile identified candidate genes related to hand osteoarthritis
Source: Arthritis Res Ther. 2021 Mar 10;23:81. doi: 10.1186/s13075-021-02458-2 (PMC7948369; doi:10.1186/s13075-021-02458-2)
Supplement: Supplementary file 1 — Additional file 1: Supplementary Table1. TWAS results of hand osteoarthritis. [file 13075_2021_2458_MOESM1_ESM.docx]

Supplementary Table1: TWAS results of hand osteoarthritis

| TISSUE | GENE | CHR | TWAS.Z | TWAS.P |
| --- | --- | --- | --- | --- |
| Muscle skeleton | ANKRD44 | 2 | 3.8381 | 0.0001 |
|  | RIC3 | 11 | 3.6172 | 0.0003 |
|  | AC005154.6 | 7 | 3.5112 | 0.0004 |
|  | PARD3 | 10 | 3.4060 | 0.0007 |
|  | GGCT | 7 | 3.3363 | 0.0008 |
|  | CRHR1 | 17 | 3.2669 | 0.0011 |
|  | LRRC37A4P | 17 | -3.2288 | 0.0012 |
|  | CRHR1-IT1 | 17 | 3.2217 | 0.0013 |
|  | KANSL1-AS1 | 17 | 3.2011 | 0.0014 |
|  | AC006547.15 | 22 | 3.1767 | 0.0015 |
|  | SRD5A3-AS1 | 4 | -3.1606 | 0.0016 |
|  | NQO2 | 6 | 3.1249 | 0.0018 |
|  | LRRC37A2 | 17 | 3.1064 | 0.0019 |
|  | TOMM22 | 22 | 3.0963 | 0.0020 |
|  | FASTKD1 | 2 | -2.9872 | 0.0028 |
|  | RAB7L1 | 1 | 2.9540 | 0.0031 |
|  | UBXN6 | 19 | -2.9523 | 0.0032 |
|  | SYVN1 | 11 | 2.9207 | 0.0035 |
|  | NCKIPSD | 3 | -2.8653 | 0.0042 |
|  | COLCA2 | 11 | 2.8613 | 0.0042 |
|  | HOTAIRM1 | 7 | -2.8422 | 0.0045 |
|  | PLEKHM1 | 17 | 2.8137 | 0.0049 |
|  | PLEKHA4 | 19 | -2.7637 | 0.0057 |
|  | L2HGDH | 14 | -2.7615 | 0.0058 |
|  | SCAMP5 | 15 | -2.7579 | 0.0058 |
|  | MXI1 | 10 | -2.7515 | 0.0059 |
|  | SPATA33 | 16 | 2.7374 | 0.0062 |
|  | POLE | 12 | -2.7238 | 0.0065 |
|  | XPA | 9 | 2.6783 | 0.0074 |
|  | CCDC13 | 3 | 2.6690 | 0.0076 |
|  | ZCCHC9 | 5 | 2.6580 | 0.0079 |
|  | RPS26 | 12 | -2.6499 | 0.0081 |
|  | RP11-98I9.4 | 6 | 2.6415 | 0.0083 |
|  | DOC2GP | 11 | -2.6353 | 0.0084 |
|  | HSD17B1 | 17 | 2.6304 | 0.0085 |
|  | C12orf73 | 12 | -2.6201 | 0.0088 |
|  | SUOX | 12 | 2.6187 | 0.0088 |
|  | SRD5A3 | 4 | -2.6181 | 0.0088 |
|  | MIR3936 | 5 | -2.6168 | 0.0089 |
|  | C5orf27 | 5 | 2.6133 | 0.0090 |
|  | ZNF107 | 7 | 2.6125 | 0.0090 |
|  | MAPKAPK5 | 12 | -2.5945 | 0.0095 |
|  | TCTEX1D1 | 1 | 2.5915 | 0.0096 |
|  | PARP14 | 3 | -2.5848 | 0.0097 |
|  | TRPM7 | 15 | -2.5844 | 0.0098 |
|  | ICA1L | 2 | -2.5772 | 0.0100 |
|  | ATP5S | 14 | -2.5768 | 0.0100 |
|  | SLC36A1 | 5 | 2.5722 | 0.0101 |
|  | L3HYPDH | 14 | 2.5665 | 0.0103 |
|  | RP11-305E17.6 | 1 | -2.5544 | 0.0106 |
|  | POLR1E | 9 | -2.5423 | 0.0110 |
|  | SMARCB1 | 22 | -2.5394 | 0.0111 |
|  | USP30-AS1 | 12 | -2.5287 | 0.0115 |
|  | TBC1D23 | 3 | 2.5186 | 0.0118 |
|  | DYNC1I1 | 7 | -2.5014 | 0.0124 |
|  | SLC51A | 3 | 2.4925 | 0.0127 |
|  | CHAF1A | 19 | -2.4715 | 0.0135 |
|  | PSMG3-AS1 | 7 | -2.4534 | 0.0142 |
|  | RBP1 | 3 | 2.4515 | 0.0142 |
|  | ZNF781 | 19 | 2.4509 | 0.0143 |
|  | CTDP1 | 18 | 2.4481 | 0.0144 |
|  | CAMLG | 5 | 2.4442 | 0.0145 |
|  | DERL3 | 22 | -2.4422 | 0.0146 |
|  | PGAM5 | 12 | -2.4399 | 0.0147 |
|  | SEMA4B | 15 | 2.4307 | 0.0151 |
|  | GTF2E1 | 3 | -2.4272 | 0.0152 |
|  | PRPSAP2 | 17 | 2.4260 | 0.0153 |
|  | AC007292.3 | 19 | 2.4244 | 0.0153 |
|  | ACOT1 | 14 | 2.4168 | 0.0157 |
|  | MPPED2 | 11 | -2.4036 | 0.0162 |
|  | FANCG | 9 | 2.3798 | 0.0173 |
|  | CCDC127 | 5 | 2.3768 | 0.0175 |
|  | CTB-50L17.9 | 19 | -2.3767 | 0.0175 |
|  | TRAPPC12-AS1 | 2 | -2.3636 | 0.0181 |
|  | PGM2 | 4 | -2.3606 | 0.0182 |
|  | IARS | 9 | 2.3602 | 0.0183 |
|  | LETMD1 | 12 | -2.3560 | 0.0185 |
|  | ZNF649 | 19 | -2.3557 | 0.0185 |
|  | CEP68 | 2 | 2.3552 | 0.0185 |
|  | RP11-932O9.7 | 15 | -2.3505 | 0.0188 |
|  | MRPS34 | 16 | 2.3464 | 0.0190 |
|  | NUS1 | 6 | -2.3401 | 0.0193 |
|  | AKR1C2 | 10 | -2.3347 | 0.0196 |
|  | RP11-767C1.2 | 3 | -2.3333 | 0.0196 |
|  | 3-Mar | 5 | 2.3272 | 0.0200 |
|  | NBN | 8 | 2.3251 | 0.0201 |
|  | CTC-429P9.2 | 19 | 2.3238 | 0.0201 |
|  | HDDC2 | 6 | -2.3188 | 0.0204 |
|  | CDH23 | 10 | -2.3181 | 0.0204 |
|  | AHSA2 | 2 | -2.3114 | 0.0208 |
|  | SAT2 | 17 | -2.3059 | 0.0211 |
|  | RP11-345P4.10 | 1 | -2.2984 | 0.0215 |
|  | RP11-138P22.1 | 17 | -2.2975 | 0.0216 |
|  | HMOX2 | 16 | 2.2878 | 0.0222 |
|  | TFCP2 | 12 | 2.2861 | 0.0223 |
|  | STYX | 14 | 2.2772 | 0.0228 |
|  | MAPT | 17 | -2.2765 | 0.0228 |
|  | PNMA1 | 14 | 2.2527 | 0.0243 |
|  | ZNF517 | 8 | 2.2518 | 0.0243 |
|  | LRRC37A | 17 | 2.2511 | 0.0244 |
|  | RP11-152F13.7 | 15 | 2.2502 | 0.0244 |
|  | CTD-3018O17.3 | 19 | -2.2456 | 0.0247 |
|  | ZNF302 | 19 | -2.2295 | 0.0258 |
|  | SNX27 | 1 | 2.2273 | 0.0259 |
|  | RP11-134G8.8 | 1 | 2.2193 | 0.0265 |
|  | TBC1D9B | 5 | -2.2056 | 0.0274 |
|  | SERPINF2 | 17 | -2.2047 | 0.0275 |
|  | CYB5D2 | 17 | -2.2035 | 0.0276 |
|  | ZSWIM7 | 17 | -2.2002 | 0.0278 |
|  | ZNRF2P2 | 7 | -2.1999 | 0.0278 |
|  | SETD6 | 16 | -2.1994 | 0.0279 |
|  | ADORA2B | 17 | -2.1989 | 0.0279 |
|  | DDX31 | 9 | -2.1990 | 0.0279 |
|  | RP11-247L20.4 | 14 | -2.1979 | 0.0280 |
|  | RPS17 | 15 | -2.1971 | 0.0280 |
|  | MZT1 | 13 | -2.1930 | 0.0283 |
|  | EIF2B2 | 14 | -2.1857 | 0.0288 |
|  | FYCO1 | 3 | -2.1814 | 0.0292 |
|  | OGN | 9 | 2.1757 | 0.0296 |
|  | ALOX5AP | 13 | 2.1700 | 0.0300 |
|  | SLC37A1 | 21 | -2.1661 | 0.0303 |
|  | MPND | 19 | 2.1475 | 0.0318 |
|  | LA16c-OS12.2 | 16 | -2.1459 | 0.0319 |
|  | TMEM185B | 2 | 2.1453 | 0.0319 |
|  | RPL23AP7 | 2 | -2.1437 | 0.0321 |
|  | FILIP1 | 6 | -2.1393 | 0.0324 |
|  | MTHFD1 | 14 | -2.1362 | 0.0327 |
|  | C15orf38 | 15 | 2.1278 | 0.0334 |
|  | FRAS1 | 4 | 2.1202 | 0.0340 |
|  | TPBG | 6 | 2.1151 | 0.0344 |
|  | ITIH4-AS1 | 3 | 2.1148 | 0.0345 |
|  | WASF3 | 13 | -2.1111 | 0.0348 |
|  | ACSM5 | 16 | -2.1005 | 0.0357 |
|  | OBFC1 | 10 | 2.0981 | 0.0359 |
|  | CTD-2203K17.1 | 5 | -2.0960 | 0.0361 |
|  | PTBP3 | 9 | 2.0913 | 0.0365 |
|  | ALG8 | 11 | 2.0878 | 0.0368 |
|  | GCC2 | 2 | 2.0855 | 0.0370 |
|  | SERPINF1 | 17 | -2.0835 | 0.0372 |
|  | FBF1 | 17 | -2.0784 | 0.0377 |
|  | TOM1 | 22 | -2.0784 | 0.0377 |
|  | CCT8 | 21 | -2.0775 | 0.0378 |
|  | ZNF839 | 14 | 2.0768 | 0.0378 |
|  | GCNT4 | 5 | 2.0739 | 0.0381 |
|  | TMEM110 | 3 | 2.0732 | 0.0382 |
|  | UNC45A | 15 | 2.0712 | 0.0384 |
|  | SNAP29 | 22 | 2.0670 | 0.0387 |
|  | THEM4 | 1 | 2.0654 | 0.0389 |
|  | AC093642.3 | 2 | -2.0643 | 0.0390 |
|  | EXOC4 | 7 | 2.0632 | 0.0391 |
|  | SCARB1 | 12 | 2.0591 | 0.0395 |
|  | MUT | 6 | 2.0569 | 0.0397 |
|  | PAOX | 10 | 2.0558 | 0.0398 |
|  | AC007308.7 | 22 | 2.0524 | 0.0401 |
|  | SLC46A3 | 13 | -2.0472 | 0.0406 |
|  | C14orf79 | 14 | -2.0474 | 0.0406 |
|  | AC108004.3 | 17 | 2.0352 | 0.0418 |
|  | DNAJB11 | 3 | -2.0331 | 0.0420 |
|  | RP1-283E3.4 | 1 | 2.0313 | 0.0422 |
|  | RP1-90J20.11 | 6 | 2.0302 | 0.0423 |
|  | GSTO2 | 10 | 2.0249 | 0.0429 |
|  | C2orf49 | 2 | -2.0247 | 0.0429 |
|  | C17orf97 | 17 | 2.0204 | 0.0433 |
|  | PPA2 | 4 | 2.0192 | 0.0435 |
|  | SLC22A5 | 5 | 2.0165 | 0.0438 |
|  | ELK4 | 1 | 2.0093 | 0.0445 |
|  | FBXO8 | 4 | 2.0075 | 0.0447 |
|  | POM121B | 7 | -2.0041 | 0.0451 |
|  | JHDM1D-AS1 | 7 | 1.9971 | 0.0458 |
|  | RP11-932O9.9 | 15 | -1.9955 | 0.0460 |
|  | HIATL1 | 9 | -1.9895 | 0.0467 |
|  | CCNT2 | 2 | 1.9888 | 0.0467 |
|  | MRPL36 | 5 | 1.9793 | 0.0478 |
|  | POGZ | 1 | 1.9734 | 0.0485 |
|  | IKBKAP | 9 | -1.9659 | 0.0493 |
|  | ZNF514 | 2 | 1.9633 | 0.0496 |
|  | GRTP1 | 13 | -1.9600 | 0.0500 |
| Peripheral blood | CRIF3:I367 | 2 | -3.6781 | 0.0002 |
|  | ZNF880 | 19 | 3.6694 | 0.0002 |
|  | NCKIPSD | 3 | -3.6290 | 0.0003 |
|  | CCR3 | 3 | -3.5323 | 0.0004 |
|  | R3HCC1 | 8 | 3.5037 | 0.0005 |
|  | RAB40C | 16 | 3.4175 | 0.0006 |
|  | TCF7 | 5 | -3.4167 | 0.0006 |
|  | SPCS1 | 3 | 3.3790 | 0.0007 |
|  | DND1 | 17 | 3.3137 | 0.0009 |
|  | NOL12 | 22 | 3.2895 | 0.0010 |
|  | SH2B3 | 12 | -3.2777 | 0.0011 |
|  | ASCC1 | 10 | -3.2727 | 0.0011 |
|  | IL18R1 | 2 | -3.2400 | 0.0012 |
|  | C6orf115 | 6 | 3.2292 | 0.0012 |
|  | SLC22A5 | 5 | 3.1584 | 0.0016 |
|  | FAH | 15 | 3.1583 | 0.0016 |
|  | TLR5 | 1 | 3.1451 | 0.0017 |
|  | SKP1 | 5 | -3.1423 | 0.0017 |
|  | TDRD9 | 14 | 3.1254 | 0.0018 |
|  | PRKACB | 1 | 3.0921 | 0.0020 |
|  | WIBG | 12 | 3.0773 | 0.0021 |
|  | LSM6 | 4 | -3.0588 | 0.0022 |
|  | CCR2 | 3 | -3.0480 | 0.0023 |
|  | KCNJ1 | 11 | -3.0457 | 0.0023 |
|  | RAB7L1 | 1 | 3.0464 | 0.0023 |
|  | PROK2 | 3 | -3.0174 | 0.0026 |
|  | EPRS | 1 | 3.0133 | 0.0026 |
|  | ZNF514 | 2 | 3.0085 | 0.0026 |
|  | NT5DC2 | 3 | -2.9948 | 0.0027 |
|  | JMJD4 | 1 | 2.9933 | 0.0028 |
|  | PUM2 | 2 | -2.9934 | 0.0028 |
|  | ANKK1 | 11 | -2.9802 | 0.0029 |
|  | FLI1 | 11 | -2.9720 | 0.0030 |
|  | FASTKD1 | 2 | -2.9690 | 0.0030 |
|  | KIAA1267 | 17 | 2.9679 | 0.0030 |
|  | DYRK4 | 12 | -2.9435 | 0.0033 |
|  | ATP6V1A | 3 | 2.9160 | 0.0035 |
|  | SRP19 | 5 | 2.9133 | 0.0036 |
|  | P4HTM | 3 | -2.8953 | 0.0038 |
|  | CD44 | 11 | 2.8406 | 0.0045 |
|  | NUCKS1 | 1 | -2.8364 | 0.0046 |
|  | KIAA0174 | 16 | -2.8348 | 0.0046 |
|  | AFF3 | 2 | -2.8235 | 0.0048 |
|  | RNF149 | 2 | -2.8236 | 0.0048 |
|  | TDRD9 | 14 | 2.8237 | 0.0048 |
|  | KIAA0494 | 1 | 2.8216 | 0.0048 |
|  | SEH1L | 18 | -2.8130 | 0.0049 |
|  | HSPBAP1 | 3 | 2.8062 | 0.0050 |
|  | USP45 | 6 | -2.7955 | 0.0052 |
|  | RRM1 | 11 | -2.7921 | 0.0052 |
|  | TM7SF2 | 11 | 2.7909 | 0.0053 |
|  | RAB7L1 | 1 | 2.7901 | 0.0053 |
|  | ADAM15 | 1 | -2.7846 | 0.0054 |
|  | SPAG7 | 17 | -2.7828 | 0.0054 |
|  | REEP5 | 5 | 2.7816 | 0.0054 |
|  | HERPUD2 | 7 | 2.7764 | 0.0055 |
|  | SFTPD | 10 | -2.7651 | 0.0057 |
|  | IL15 | 4 | -2.7561 | 0.0059 |
|  | CASP1 | 11 | 2.7538 | 0.0059 |
|  | TMEM169 | 2 | 2.7368 | 0.0062 |
|  | QRICH1 | 3 | 2.7170 | 0.0066 |
|  | PSMD2 | 3 | -2.7156 | 0.0066 |
|  | EIF3F | 11 | 2.7138 | 0.0067 |
|  | SLC22A5 | 5 | 2.7138 | 0.0067 |
|  | DDX17 | 22 | -2.7126 | 0.0067 |
|  | MTRF1 | 13 | -2.7024 | 0.0069 |
|  | DHRS3 | 1 | -2.6988 | 0.0070 |
|  | FEZ2 | 2 | 2.6912 | 0.0071 |
|  | FCER1G | 1 | -2.6884 | 0.0072 |
|  | TNNI2 | 11 | 2.6752 | 0.0075 |
|  | TAGLN2 | 1 | -2.6679 | 0.0076 |
|  | GTF2E2 | 8 | -2.6677 | 0.0076 |
|  | ABI3 | 17 | 2.6643 | 0.0077 |
|  | DUSP16 | 12 | -2.6628 | 0.0078 |
|  | FAU | 11 | 2.6590 | 0.0078 |
|  | VPS11 | 11 | -2.6549 | 0.0079 |
|  | RNF182 | 6 | 2.6540 | 0.0080 |
|  | C5orf56 | 5 | 2.6528 | 0.0080 |
|  | STRN4 | 19 | -2.6489 | 0.0081 |
|  | HERPUD1 | 16 | -2.6476 | 0.0081 |
|  | FCER1G | 1 | -2.6428 | 0.0082 |
|  | ARSB | 5 | -2.6431 | 0.0082 |
|  | WBSCR22 | 7 | 2.6414 | 0.0083 |
|  | AGBL3 | 7 | 2.6414 | 0.0083 |
|  | PDXP | 22 | -2.6369 | 0.0084 |
|  | RAD51C | 17 | -2.6350 | 0.0084 |
|  | ACOX1 | 17 | 2.6325 | 0.0085 |
|  | ANKDD1A | 15 | 2.6277 | 0.0086 |
|  | C2orf74 | 2 | 2.6252 | 0.0087 |
|  | KAT2B | 3 | -2.6155 | 0.0089 |
|  | GNRHR | 4 | 2.6130 | 0.0090 |
|  | TMPRSS11D | 4 | 2.6130 | 0.0090 |
|  | ANKH | 5 | -2.6074 | 0.0091 |
|  | RCOR1 | 14 | 2.6010 | 0.0093 |
|  | WDR6 | 3 | 2.5958 | 0.0094 |
|  | CARD16 | 11 | 2.5954 | 0.0095 |
|  | DPY19L2 | 12 | 2.5914 | 0.0096 |
|  | DCBLD1 | 6 | 2.5909 | 0.0096 |
|  | METAP2 | 12 | -2.5895 | 0.0096 |
|  | EBF4 | 20 | -2.5873 | 0.0097 |
|  | HEATR4 | 14 | 2.5862 | 0.0097 |
|  | PTOV1 | 19 | -2.5820 | 0.0098 |
|  | ALDH3A2 | 17 | 2.5760 | 0.0100 |
|  | MPP2 | 17 | 2.5760 | 0.0100 |
|  | PLEKHM1 | 17 | 2.5760 | 0.0100 |
|  | NSUN2 | 5 | 2.5751 | 0.0100 |
|  | C17orf51 | 17 | 2.5652 | 0.0103 |
|  | TIPARP | 3 | -2.5636 | 0.0104 |
|  | GRPEL2 | 5 | -2.5593 | 0.0105 |
|  | TCTA | 3 | -2.5569 | 0.0106 |
|  | VPS8 | 3 | 2.5549 | 0.0106 |
|  | JMJD8 | 16 | 2.5543 | 0.0106 |
|  | THEM4 | 1 | 2.5522 | 0.0107 |
|  | SLC35B4 | 7 | 2.5464 | 0.0109 |
|  | USP3 | 15 | -2.5363 | 0.0112 |
|  | ACACB | 12 | -2.5360 | 0.0112 |
|  | IARS | 9 | -2.5335 | 0.0113 |
|  | ZNF514 | 2 | 2.5294 | 0.0114 |
|  | C2CD2 | 21 | 2.5280 | 0.0115 |
|  | TUBG2 | 17 | -2.5259 | 0.0115 |
|  | MAP1LC3B | 16 | -2.5225 | 0.0117 |
|  | CIB1 | 15 | 2.5216 | 0.0117 |
|  | ACTG1 | 17 | 2.5212 | 0.0117 |
|  | PANK2 | 20 | 2.5184 | 0.0118 |
|  | ADORA3 | 1 | -2.5179 | 0.0118 |
|  | ATP5S | 14 | -2.5176 | 0.0118 |
|  | MTRF1 | 13 | -2.5122 | 0.0120 |
|  | ZNF793 | 19 | -2.5116 | 0.0120 |
|  | RCN3 | 19 | -2.5088 | 0.0121 |
|  | ZNF302 | 19 | -2.5067 | 0.0122 |
|  | ZNF480 | 19 | -2.5010 | 0.0124 |
|  | SAMD12 | 8 | -2.4985 | 0.0125 |
|  | UBXN6 | 19 | -2.4933 | 0.0127 |
|  | LYRM7 | 5 | -2.4925 | 0.0127 |
|  | UBE2F | 2 | 2.4901 | 0.0128 |
|  | MBP | 18 | -2.4872 | 0.0129 |
|  | HIPK2 | 7 | -2.4878 | 0.0129 |
|  | SPRED1 | 15 | -2.4855 | 0.0129 |
|  | KLC1 | 14 | -2.4833 | 0.0130 |
|  | DDX10 | 11 | 2.4828 | 0.0130 |
|  | CLOCK | 4 | -2.4805 | 0.0131 |
|  | DBNL | 7 | -2.4759 | 0.0133 |
|  | TRAP1 | 16 | -2.4696 | 0.0135 |
|  | IDH2 | 15 | -2.4694 | 0.0135 |
|  | STAB1 | 3 | 2.4683 | 0.0136 |
|  | BZW2 | 7 | -2.4688 | 0.0136 |
|  | TMEM40 | 3 | 2.4651 | 0.0137 |
|  | CIB1 | 15 | 2.4567 | 0.0140 |
|  | DCBLD1 | 6 | 2.4560 | 0.0140 |
|  | SAR1A | 10 | 2.4553 | 0.0141 |
|  | GNL3 | 3 | -2.4525 | 0.0142 |
|  | EFR3A | 8 | 2.4524 | 0.0142 |
|  | ENDOG | 9 | 2.4518 | 0.0142 |
| YBL | ADAM8 | 10 | 2.4523 | 0.0142 |
|  | NFKB1 | 4 | -2.4475 | 0.0144 |
|  | PDE8B | 5 | 2.4428 | 0.0146 |
|  | XPA | 9 | -2.4424 | 0.0146 |
|  | CASP5 | 11 | 2.4418 | 0.0146 |
|  | SFXN5 | 2 | 2.4373 | 0.0148 |
|  | BLK | 8 | 2.4363 | 0.0148 |
|  | NBN | 8 | -2.4310 | 0.0151 |
|  | AZI1 | 17 | 2.4305 | 0.0151 |
|  | RAD51C | 17 | -2.4300 | 0.0151 |
|  | ENO1 | 1 | -2.4277 | 0.0152 |
|  | SYK | 9 | -2.4275 | 0.0152 |
|  | ALDH6A1 | 14 | -2.4208 | 0.0155 |
|  | DYNLT1 | 6 | 2.4201 | 0.0155 |
|  | IGSF22 | 11 | 2.4151 | 0.0157 |
|  | PEF1 | 1 | 2.4123 | 0.0159 |
|  | SLC20A1 | 2 | 2.4031 | 0.0163 |
|  | ARIH2 | 3 | -2.3946 | 0.0166 |
|  | GATS | 7 | -2.3903 | 0.0168 |
|  | FAM43A | 3 | -2.3894 | 0.0169 |
|  | SETDB1 | 1 | 2.3888 | 0.0169 |
|  | SEMA4A | 1 | 2.3869 | 0.0170 |
|  | ACTR5 | 20 | -2.3858 | 0.0170 |
|  | ARL17B | 17 | -2.3842 | 0.0171 |
|  | CYB561 | 17 | 2.3812 | 0.0173 |
|  | FBXO18 | 10 | 2.3770 | 0.0175 |
|  | SLC16A6 | 17 | -2.3758 | 0.0175 |
|  | FNTB | 14 | -2.3749 | 0.0176 |
|  | CASP1 | 11 | 2.3749 | 0.0176 |
|  | IRF4 | 6 | 2.3721 | 0.0177 |
|  | LPXN | 11 | 2.3698 | 0.0178 |
|  | ZNHIT6 | 1 | 2.3640 | 0.0181 |
|  | IL17RA | 22 | -2.3640 | 0.0181 |
|  | FADS1 | 11 | 2.3632 | 0.0181 |
|  | CECR6 | 22 | 2.3548 | 0.0185 |
|  | NDFIP1 | 5 | 2.3495 | 0.0188 |
|  | INTS1 | 7 | -2.3490 | 0.0188 |
|  | GATAD1 | 7 | -2.3500 | 0.0188 |
|  | SUOX | 12 | 2.3448 | 0.0190 |
|  | SIPA1L2 | 1 | 2.3420 | 0.0192 |
|  | ZSWIM7 | 17 | 2.3414 | 0.0192 |
|  | APBA2 | 15 | -2.3372 | 0.0194 |
|  | DAPK1 | 9 | -2.3340 | 0.0196 |
|  | GAL3ST4 | 7 | -2.3319 | 0.0197 |
|  | IRF1 | 5 | -2.3278 | 0.0199 |
|  | PRUNE | 1 | 2.3264 | 0.0200 |
|  | CHN2 | 7 | -2.3263 | 0.0200 |
|  | ADORA2B | 17 | -2.3260 | 0.0200 |
|  | ELF2 | 4 | 2.3257 | 0.0200 |
|  | LARGE | 22 | -2.3249 | 0.0201 |
|  | PEX5 | 12 | -2.3210 | 0.0203 |
|  | PARK7 | 1 | -2.3196 | 0.0204 |
|  | DLL1 | 6 | -2.3175 | 0.0205 |
|  | SRD5A3 | 4 | -2.3150 | 0.0206 |
|  | CHN2 | 7 | -2.3118 | 0.0208 |
|  | NQO2 | 6 | 2.3083 | 0.0210 |
|  | ST3GAL3 | 1 | 2.3067 | 0.0211 |
|  | SLC22A4 | 5 | 2.3035 | 0.0212 |
|  | CXCR6 | 3 | -2.3035 | 0.0213 |
|  | BAMBI | 10 | -2.3032 | 0.0213 |
|  | RAPGEF1 | 9 | -2.3024 | 0.0213 |
|  | IARS | 9 | -2.3008 | 0.0214 |
|  | DHX34 | 19 | -2.2971 | 0.0216 |
|  | FAM160A1 | 4 | 2.2969 | 0.0216 |
|  | PEX13 | 2 | 2.2926 | 0.0219 |
|  | ZNF622 | 5 | -2.2909 | 0.0220 |
|  | USP4 | 3 | 2.2887 | 0.0221 |
|  | LACTB2 | 8 | -2.2839 | 0.0224 |
|  | ZMYM5 | 13 | 2.2815 | 0.0225 |
|  | PALM2 | 9 | -2.2792 | 0.0227 |
|  | NICN1 | 3 | 2.2782 | 0.0227 |
|  | CHN1 | 2 | 2.2767 | 0.0228 |
|  | ACOX1 | 17 | 2.2742 | 0.0230 |
|  | DIRC2 | 3 | 2.2722 | 0.0231 |
|  | CCT8 | 21 | 2.2722 | 0.0231 |
|  | PSMB4 | 1 | -2.2686 | 0.0233 |
|  | HERPUD1 | 16 | -2.2680 | 0.0233 |
|  | TMUB2 | 17 | 2.2678 | 0.0233 |
|  | FASTKD2 | 2 | -2.2663 | 0.0234 |
|  | IER5 | 1 | 2.2654 | 0.0235 |
|  | DFNB31 | 9 | 2.2612 | 0.0237 |
|  | DDX5 | 17 | -2.2572 | 0.0240 |
|  | BAZ2A | 12 | -2.2564 | 0.0241 |
|  | KIAA0232 | 4 | -2.2529 | 0.0243 |
|  | CLEC12A | 12 | -2.2484 | 0.0246 |
|  | PSTK | 10 | -2.2480 | 0.0246 |
|  | SCARB1 | 12 | 2.2468 | 0.0247 |
|  | STX8 | 17 | -2.2431 | 0.0249 |
|  | CFD | 19 | -2.2428 | 0.0249 |
|  | SH3TC1 | 4 | -2.2427 | 0.0249 |
|  | EXT1 | 8 | -2.2423 | 0.0249 |
|  | HDHD2 | 18 | 2.2394 | 0.0251 |
|  | PPP1R15A | 19 | 2.2334 | 0.0255 |
|  | TTC39B | 9 | -2.2291 | 0.0258 |
|  | SLC22A4 | 5 | 2.2291 | 0.0258 |
|  | DDX5 | 17 | -2.2278 | 0.0259 |
|  | TMEM175 | 4 | 2.2240 | 0.0262 |
|  | PVRIG | 7 | -2.2235 | 0.0262 |
|  | BLOC1S2 | 10 | 2.2224 | 0.0263 |
|  | RNF13 | 3 | 2.2172 | 0.0266 |
|  | PKHD1L1 | 8 | -2.2159 | 0.0267 |
|  | ZNF524 | 19 | 2.2122 | 0.0270 |
|  | PPFIA4 | 1 | -2.2114 | 0.0270 |
|  | NEBL | 10 | -2.2095 | 0.0271 |
|  | CD160 | 1 | -2.2064 | 0.0274 |
|  | DIXDC1 | 11 | 2.2038 | 0.0275 |
|  | SAT2 | 17 | -2.1990 | 0.0279 |
|  | POLG2 | 17 | 2.1979 | 0.0280 |
|  | ERGIC1 | 5 | -2.1952 | 0.0282 |
|  | NUCB1 | 19 | 2.1923 | 0.0284 |
|  | MYST3 | 8 | 2.1917 | 0.0284 |
|  | RPL36 | 19 | -2.1917 | 0.0284 |
|  | UBQLN4 | 1 | -2.1910 | 0.0285 |
|  | ABHD2 | 15 | 2.1907 | 0.0285 |
|  | CDK11A | 1 | 2.1832 | 0.0290 |
|  | CLCN6 | 1 | 2.1821 | 0.0291 |
|  | SYNGR1 | 22 | -2.1788 | 0.0294 |
|  | CD44 | 11 | 2.1755 | 0.0296 |
|  | PGGT1B | 5 | -2.1752 | 0.0296 |
|  | GNG2 | 14 | 2.1746 | 0.0297 |
|  | SERGEF | 11 | 2.1704 | 0.0300 |
|  | PSMD1 | 2 | -2.1645 | 0.0304 |
|  | NBL1 | 1 | 2.1630 | 0.0305 |
|  | MLKL | 16 | 2.1628 | 0.0306 |
|  | ACAT2 | 6 | 2.1624 | 0.0306 |
|  | TTC39B | 9 | -2.1616 | 0.0306 |
|  | BCS1L | 2 | 2.1536 | 0.0313 |
|  | MAP2K3 | 17 | -2.1499 | 0.0316 |
|  | GCC2 | 2 | 2.1467 | 0.0318 |
|  | MON1B | 16 | 2.1439 | 0.0320 |
|  | PRPSAP2 | 17 | -2.1435 | 0.0321 |
|  | SIPA1L2 | 1 | 2.1430 | 0.0321 |
|  | ALCAM | 3 | -2.1426 | 0.0321 |
|  | CC2D2A | 4 | 2.1420 | 0.0322 |
|  | SYK | 9 | -2.1419 | 0.0322 |
|  | ANKFY1 | 17 | -2.1417 | 0.0322 |
|  | ST6GALNAC3 | 1 | -2.1414 | 0.0322 |
|  | TANC2 | 17 | -2.1394 | 0.0324 |
|  | ADAM28 | 8 | -2.1386 | 0.0325 |
|  | LILRA5 | 19 | 2.1382 | 0.0325 |
|  | ACOT4 | 14 | 2.1357 | 0.0327 |
|  | FAM8A1 | 6 | 2.1336 | 0.0329 |
|  | GKAP1 | 9 | 2.1313 | 0.0331 |
|  | IRS2 | 13 | -2.1303 | 0.0331 |
|  | CTBS | 1 | -2.1278 | 0.0334 |
|  | RARS2 | 6 | -2.1268 | 0.0334 |
|  | TCN2 | 22 | 2.1247 | 0.0336 |
|  | SUCLA2 | 13 | 2.1238 | 0.0337 |
|  | FBXO3 | 11 | 2.1193 | 0.0341 |
|  | C15orf38 | 15 | -2.1175 | 0.0342 |
|  | ZBTB38 | 3 | -2.1167 | 0.0343 |
|  | RASSF2 | 20 | 2.1154 | 0.0344 |
|  | COPG2 | 7 | 2.1151 | 0.0344 |
|  | LILRA3 | 19 | 2.1147 | 0.0345 |
|  | MAP1LC3B | 16 | -2.1111 | 0.0348 |
|  | PGM2 | 4 | -2.1109 | 0.0348 |
|  | MED6 | 14 | 2.1103 | 0.0348 |
|  | RNF13 | 3 | 2.1099 | 0.0349 |
|  | OSGIN2 | 8 | 2.1076 | 0.0351 |
|  | PASK | 2 | -2.1042 | 0.0354 |
|  | N6AMT2 | 13 | -2.1035 | 0.0354 |
|  | NFATC1 | 18 | -2.1013 | 0.0356 |
|  | FRMD3 | 9 | 2.1004 | 0.0357 |
|  | MRPL36 | 5 | 2.0963 | 0.0361 |
|  | SLC4A2 | 7 | -2.0955 | 0.0361 |
|  | ATF5 | 19 | -2.0950 | 0.0362 |
|  | H2AFY2 | 10 | -2.0948 | 0.0362 |
|  | COMMD5 | 8 | -2.0927 | 0.0364 |
|  | ATP6V0E2 | 7 | -2.0930 | 0.0364 |
|  | EIF3M | 11 | -2.0923 | 0.0364 |
|  | P2RX1 | 17 | -2.0912 | 0.0365 |
|  | RBMS2 | 12 | -2.0910 | 0.0365 |
|  | BACH2 | 6 | 2.0901 | 0.0366 |
|  | TBRG4 | 7 | 2.0871 | 0.0369 |
|  | AHSA2 | 2 | -2.0867 | 0.0369 |
|  | L2HGDH | 14 | 2.0840 | 0.0372 |
|  | GPX3 | 5 | -2.0772 | 0.0378 |
|  | IL18R1 | 2 | -2.0743 | 0.0381 |
|  | CDC34 | 19 | 2.0707 | 0.0384 |
|  | CARD16 | 11 | 2.0699 | 0.0385 |
|  | KCNAB1 | 3 | -2.0690 | 0.0385 |
|  | RARS | 5 | -2.0674 | 0.0387 |
|  | PPIA | 7 | 2.0655 | 0.0389 |
|  | SYNGR1 | 22 | -2.0645 | 0.0390 |
|  | PRR11 | 17 | -2.0643 | 0.0390 |
|  | FADS2 | 11 | 2.0641 | 0.0390 |
|  | EGLN3 | 14 | -2.0635 | 0.0391 |
|  | TAX1BP3 | 17 | -2.0617 | 0.0392 |
|  | RSPH3 | 6 | -2.0608 | 0.0393 |
|  | SEMA4A | 1 | 2.0595 | 0.0394 |
|  | TLR5 | 1 | 2.0596 | 0.0394 |
|  | ZNF138 | 7 | -2.0540 | 0.0400 |
|  | PPFIA4 | 1 | -2.0535 | 0.0400 |
|  | TRBC2 | 7 | -2.0532 | 0.0401 |
|  | AK5 | 1 | 2.0523 | 0.0401 |
|  | PELI1 | 2 | 2.0500 | 0.0404 |
|  | DOK6 | 18 | 2.0471 | 0.0406 |
|  | HEXDC | 17 | -2.0470 | 0.0407 |
|  | SIN3B | 19 | 2.0469 | 0.0407 |
|  | HOXA9 | 7 | 2.0457 | 0.0408 |
|  | RNASE3 | 14 | 2.0385 | 0.0415 |
|  | PHLPP2 | 16 | 2.0375 | 0.0416 |
|  | CFL2 | 14 | 2.0366 | 0.0417 |
|  | TGFBI | 5 | 2.0350 | 0.0419 |
|  | FAM198B | 4 | -2.0344 | 0.0419 |
|  | RNASET2 | 6 | 2.0289 | 0.0425 |
|  | ITFG2 | 12 | -2.0276 | 0.0426 |
|  | BBS10 | 12 | -2.0263 | 0.0427 |
|  | PLCL2 | 3 | -2.0262 | 0.0427 |
|  | PIGK | 1 | 2.0238 | 0.0430 |
|  | RMI1 | 9 | -2.0235 | 0.0430 |
|  | MTMR4 | 17 | 2.0217 | 0.0432 |
|  | KCNQ5 | 6 | 2.0196 | 0.0434 |
|  | FZD2 | 17 | -2.0181 | 0.0436 |
|  | FLII | 17 | -2.0180 | 0.0436 |
|  | SUN2 | 22 | -2.0175 | 0.0436 |
|  | ZNF571 | 19 | 2.0172 | 0.0437 |
|  | RNASET2 | 6 | 2.0174 | 0.0437 |
|  | NQO2 | 6 | 2.0163 | 0.0438 |
|  | SAP18 | 13 | 2.0156 | 0.0438 |
|  | IPPK | 9 | 2.0139 | 0.0440 |
|  | KAT2B | 3 | -2.0139 | 0.0440 |
|  | POMC | 2 | -2.0137 | 0.0440 |
|  | ATP8B4 | 15 | -2.0137 | 0.0440 |
|  | SPOCK2 | 10 | 2.0118 | 0.0442 |
|  | DAPK1 | 9 | -2.0120 | 0.0442 |
|  | BLOC1S2 | 10 | 2.0112 | 0.0443 |
|  | XRCC3 | 14 | -2.0102 | 0.0444 |
|  | TOM1 | 22 | -2.0077 | 0.0447 |
|  | PNPT1 | 2 | -2.0073 | 0.0447 |
|  | CHMP2B | 3 | 2.0069 | 0.0448 |
|  | DDA1 | 19 | 2.0037 | 0.0451 |
|  | ZNF491 | 19 | 2.0025 | 0.0452 |
|  | NUP88 | 17 | -2.0011 | 0.0454 |
|  | CNTNAP1 | 17 | -2.0009 | 0.0454 |
|  | EPS15L1 | 19 | 1.9997 | 0.0455 |
|  | FPR3 | 19 | -1.9992 | 0.0456 |
|  | CSRP2BP | 20 | -1.9991 | 0.0456 |
|  | TCFL5 | 20 | -1.9989 | 0.0456 |
|  | CIZ1 | 9 | 1.9968 | 0.0458 |
|  | TOB1 | 17 | 1.9969 | 0.0458 |
|  | PRDX5 | 11 | 1.9955 | 0.0460 |
|  | MLLT10 | 10 | -1.9951 | 0.0460 |
|  | DNASE2 | 19 | -1.9927 | 0.0463 |
|  | MRPL35 | 2 | -1.9906 | 0.0465 |
|  | APOL1 | 22 | 1.9876 | 0.0469 |
|  | ARL16 | 17 | -1.9835 | 0.0473 |
|  | ISCU | 12 | -1.9826 | 0.0474 |
|  | CD40 | 20 | 1.9815 | 0.0475 |
|  | SKP2 | 5 | 1.9814 | 0.0475 |
|  | FAM101B | 17 | 1.9814 | 0.0475 |
|  | MS4A14 | 11 | 1.9777 | 0.0480 |
|  | ANKRD9 | 14 | 1.9771 | 0.0480 |
|  | RPL36AL | 14 | 1.9760 | 0.0482 |
|  | ITIH4 | 3 | 1.9759 | 0.0482 |
|  | CNOT1 | 16 | -1.9754 | 0.0482 |
|  | PXK | 3 | 1.9745 | 0.0483 |
|  | BST1 | 4 | 1.9741 | 0.0484 |
|  | MS4A14 | 11 | 1.9739 | 0.0484 |
|  | DEF8 | 16 | -1.9725 | 0.0486 |
|  | PRCP | 11 | -1.9701 | 0.0488 |
|  | UBE2B | 5 | -1.9701 | 0.0488 |
|  | PASK | 2 | -1.9674 | 0.0491 |
|  | VRK2 | 2 | 1.9674 | 0.0491 |
|  | ST7 | 7 | -1.9670 | 0.0492 |
|  | PKN3 | 9 | -1.9671 | 0.0492 |
|  | CTNNB1 | 3 | 1.9651 | 0.0494 |
|  | PARK7 | 1 | -1.9645 | 0.0495 |
|  | BTNL8 | 5 | 1.9605 | 0.0499 |
|  | C2orf24 | 2 | 1.9600 | 0.0500 |

Note: The GWAS summary data for hand OA was derived from the UK Biobank (UK Biobank fields: 20002), which contains 452,264 White British individuals, including 37,782 osteoarthritis patients. The TWAS.P and TWAS.Z values were calculated by the FUSION approach (<http://gusevlab.org/projects/fusion/>),

TWAS Transcriptome-Wide Association Study, GWAS Genome-Wide Association Study, TWAS.P TWAS P value, TWAS.Z TWAS Z score.
