# Supplementary material for: Integrating transcriptome-wide association study and mRNA expression profile identified candidate genes related to hand osteoarthritis
Source: Arthritis Res Ther. 2021 Mar 10;23:81. doi: 10.1186/s13075-021-02458-2 (PMC7948369; doi:10.1186/s13075-021-02458-2)
Supplement: Supplementary file 2 — Additional file 2: Supplementary Table2. GO enrichment analysis results of candidate genes detected by TWAS of hand osteoarthritis. [file 13075_2021_2458_MOESM2_ESM.docx]

Supplementary Table2: GO enrichment analysis results of candidate genes detected by TWAS of hand osteoarthritis

| Term | P value* |
| --- | --- |
| GO:0005515~protein binding | 0.0003 |
| GO:0005829~cytosol | 0.0012 |
| GO:0016020~membrane | 0.0014 |
| GO:0002554~serotonin secretion by platelet | 0.0018 |
| GO:0005739~mitochondrion | 0.0023 |
| GO:0000038~very long-chain fatty acid metabolic process | 0.0046 |
| GO:0005524~ATP binding | 0.0049 |
| GO:0005782~peroxisomal matrix | 0.0050 |
| GO:0005759~mitochondrial matrix | 0.0064 |
| GO:0005657~replication fork | 0.0075 |
| GO:0000731~DNA synthesis involved in DNA repair | 0.0109 |
| GO:0030176~integral component of endoplasmic reticulum membrane | 0.0133 |
| GO:0002223~stimulatory C-type lectin receptor signaling pathway | 0.0163 |
| GO:0003690~double-stranded DNA binding | 0.0167 |
| GO:0034394~protein localization to cell surface | 0.0168 |
| GO:0006418~tRNA aminoacylation for protein translation | 0.0173 |
| GO:0005737~cytoplasm | 0.0183 |
| GO:0034058~endosomal vesicle fusion | 0.0200 |
| GO:0050764~regulation of phagocytosis | 0.0200 |
| GO:0061630~ubiquitin protein ligase activity | 0.0209 |
| GO:0004672~protein kinase activity | 0.0209 |
| GO:0070062~extracellular exosome | 0.0225 |
| GO:0004004~ATP-dependent RNA helicase activity | 0.0242 |
| GO:0033148~positive regulation of intracellular estrogen receptor signaling pathway | 0.0246 |
| GO:0005654~nucleoplasm | 0.0248 |
| GO:0000732~strand displacement | 0.0264 |
| GO:0006461~protein complex assembly | 0.0265 |
| GO:0070936~protein K48-linked ubiquitination | 0.0296 |
| GO:0033674~positive regulation of kinase activity | 0.0296 |
| GO:0043306~positive regulation of mast cell degranulation | 0.0296 |
| GO:0016290~palmitoyl-CoA hydrolase activity | 0.0302 |
| GO:0016491~oxidoreductase activity | 0.0312 |
| GO:0071260~cellular response to mechanical stimulus | 0.0324 |
| GO:0005758~mitochondrial intermembrane space | 0.0338 |
| GO:0006281~DNA repair | 0.0351 |
| GO:0000975~regulatory region DNA binding | 0.0356 |
| GO:0000724~double-strand break repair via homologous recombination | 0.0378 |
| GO:0036109~alpha-linolenic acid metabolic process | 0.0406 |
| GO:0005777~peroxisome | 0.0410 |
| GO:0003724~RNA helicase activity | 0.0414 |
| GO:0005778~peroxisomal membrane | 0.0417 |
| GO:0055114~oxidation-reduction process | 0.0424 |
| GO:0001741~XY body | 0.0441 |
| GO:0045595~regulation of cell differentiation | 0.0451 |
| GO:0034515~proteasome storage granule | 0.0479 |
| GO:0033065~Rad51C-XRCC3 complex | 0.0479 |
| GO:0005764~lysosome | 0.0492 |
| GO:0045204~MAPK export from nucleus | 0.0494 |
| GO:0019085~early viral transcription | 0.0494 |
| GO:0019840~isoprenoid binding | 0.0500 |
| GO:0015491~cation:cation antiporter activity | 0.0500 |

Note: the candidate genes identified by TWAS were further analyzed by the Database for Annotation, Visualization and Integrated Discovery tool (https://david.ncifcrf.gov/) for GO enrichment analysis.

GO , Gene Ontology.
