# Supplementary material for: Integrating transcriptome-wide association study and mRNA expression profile identified candidate genes related to hand osteoarthritis
Source: Arthritis Res Ther. 2021 Mar 10;23:81. doi: 10.1186/s13075-021-02458-2 (PMC7948369; doi:10.1186/s13075-021-02458-2)
Supplement: Supplementary file 3 — Additional file 3: Supplementary Table3. Pathway enrichment analysis results of candidate genes identified by the TWAS of hand osteoarthritis. [file 13075_2021_2458_MOESM3_ESM.docx]

Supplementary Table3: Pathway enrichment analysis results of candidate genes identified by the TWAS of hand osteoarthritis

| Pathway | P value |
| --- | --- |
| hsa01040:Biosynthesis of unsaturated fatty acids | 0.0034 |
| hsa00640:Propanoate metabolism | 0.0071 |
| h_pcafPathway:The information-processing pathway at the IFN-beta enhancer | 0.0074 |
| hsa05132:Salmonella infection | 0.0280 |
| h_skp2e2fPathway:E2F1 Destruction Pathway | 0.0344 |

Note: the candidate genes identified by TWAS were further analyzed by the Database for Annotation, Visualization and Integrated Discovery tool (https://david.ncifcrf.gov/) for pathway enrichment analysis.
